# Treatment burden and associated factors: a population-based survey in Central Denmark Region 2017: Marie Hauge Pedersen

**DOI:** 10.1093/eurpub/ckac131.144

**Published:** 2022-10-25

**Authors:** MH Pedersen, P Duncan, M Lasgaard, K Friis, C Salisbury, FB Larsen

**Affiliations:** 1 Public Health and Health Services Research, DEFACTUM, Central Denmark Region, Aarhus N, Denmark; 2 Centre for Academic Primary Care, NIHR School for Primary Care Research, University of Bristol, Bristol, UK

## Abstract

**Background:**

Exploring treatment burden at a population level can provide evidence of the types of patients who need special attention and support. We aimed to determine factors associated with high perceived treatment burden in a population-based survey of adults living in the Central Denmark Region (23% of the Danish population).

**Methods:**

The Danish Multimorbidity Treatment Burden Questionnaire (MTBQ) was included in the 2017 Danish population health survey. 28,627 individuals aged 25 years or over participated (64% response rate). Individuals who reported having one or more medical conditions or attending regular health check-ups were asked to complete the MTBQ. A global MTBQ score was calculated (range 0-100) and both the continuous scores and a four-category grouping of the scores into no, low, medium and high burden were used to statistically assess the association between treatment burden and sociodemographic and health-related factors.

**Results:**

13,407 individuals completed the Danish MTBQ (mean age 59 years). Treatment burden was negatively associated with self-related health (rs = -0.45, P < 0.0001), health-related quality of life (rs = -0.46/-0.51, P < 0.0001) and positively associated with the number of long-term conditions (rs = 0.26, P < 0.0001) and perceived stress (rs = 0.44, P < 0.0001). Higher treatment burden was associated with young age, male sex, high educational level, unemployment, not living with a spouse/cohabitant, living with child(ren) and specific long-term conditions, including heart disease, stroke, diabetes and mental illness.

**Conclusions:**

This is the first known population-based study of treatment burden. The findings provide important evidence to policy makers and clinicians about sociodemographic groups at risk of higher treatment burden. We recommend that patient-perceived treatment burden is included when evaluating interventions targeting patients with long-term conditions and multimorbidity and health-care system reorganisations.

**Key messages:**

• Treatment burden is associated with poor health and health-related quality of life and, among others, young age, male sex, unemployment, not living with a spouse, and specific long-term conditions.

• We recommend that patient-perceived treatment burden is included when evaluating interventions targeting patients with long-term conditions and multimorbidity and health-care system reorganisations.

